# Two Unique Ligand-Binding Clamps of *Rhizopus oryzae* Starch Binding Domain for Helical Structure Disruption of Amylose

**DOI:** 10.1371/journal.pone.0041131

**Published:** 2012-07-17

**Authors:** Ting-Ying Jiang, Yuan-Pei Ci, Wei-I Chou, Yuan-Chuan Lee, Yuh-Ju Sun, Wei-Yao Chou, Kun-Mou Li, Margaret Dah-Tsyr Chang

**Affiliations:** 1 Institute of Molecular and Cellular Biology and Department of Medical Science, National Tsing Hua University, Hsinchu, Taiwan, Republic of China; 2 Simpson Biotech Company, Ltd., Taoyuan County, Taiwan, Republic of China; 3 Department of Biology, Johns Hopkins University, Baltimore, Maryland, United States of America; 4 Institute of Bioinformatics and Structural Biology and Department of Life Science, National Tsing Hua University, Hsinchu, Taiwan, Republic of China; 5 Department of Computer Science, National Tsing Hua University, Hsinchu, Taiwan, Republic of China; Russian Academy of Sciences, Institute for Biological Instrumentation, Russian Federation

## Abstract

The *N*-terminal starch binding domain of *Rhizopus oryzae* glucoamylase (*Ro*SBD) has a high binding affinity for raw starch. *Ro*SBD has two ligand-binding sites, each containing a ligand-binding clamp: a polyN clamp residing near binding site I is unique in that it is expressed in only three members of carbohydrate binding module family 21 (CBM21) members, and a Y32/F58 clamp located at binding site II is conserved in several CBMs. Here we characterized different roles of these sites in the binding of insoluble and soluble starches using an amylose-iodine complex assay, atomic force microscopy, isothermal titration calorimetry, site-directed mutagenesis, and structural bioinformatics. *Ro*SBD induced the release of iodine from the amylose helical cavity and disrupted the helical structure of amylose type III, thereby significantly diminishing the thickness and length of the amylose type III fibrils. A point mutation in the critical ligand-binding residues of sites I and II, however, reduced both the binding affinity and amylose helix disruption. This is the first molecular model for structure disruption of the amylose helix by a non-hydrolytic CBM21 member. *Ro*SBD apparently twists the helical amylose strands apart to expose more ligand surface for further SBD binding. Repeating the process triggers the relaxation and unwinding of amylose helices to generate thinner and shorter amylose fibrils, which are more susceptible to hydrolysis by glucoamylase. This model aids in understanding the natural roles of CBMs in protein-glycan interactions and contributes to potential molecular engineering of CBMs.

## Introduction

Starch is the primary form of carbohydrate storage in plant tubers and seed endosperm [1] and is comprised of 70–80% amylopectin, which has a compact, branched molecular structure, and amylose (20–30%), which has an extended conformation. Amylose is a linear polysaccharide composed of mostly unbranched *α*-1,4-D-glucose repeating units. Amylopectin has a higher molecular weight, with *α*-1,4-D-glucose repeats and frequent α-1,6 branches [2]. Glucoamylase (GA; EC 3.2.1.3) is an enzyme capable of hydrolyzing α-1,4 glycosidic linkages from the non-reducing ends of starch and related oligosaccharides to release β-D-glucose [3]. GA from the fungus *Rhizopus oryzae* has two functional domains: an *N-*terminal starch binding domain (*Ro*SBD; residues 26–131), classified as belonging to family 21 of the carbohydrate binding modules (*Ro*CBM21), and a *C-*terminal catalytic domain (residues 168–604), classified as belonging to family 15 of the glycoside hydrolases (GH15) [4]. These two domains are joined by an *O*-glycosylated linking sequence (residues 132–167). The SBD of GA facilitates hydrolysis of raw starch by facilitating GH adsorption to the surface of starch [5].

The CBMs of carbohydrate-active enzymes have been classified in the CAZy database (http://www.cazy.org/) into 64 families based on their amino acid sequence similarity and ligand specificity [6]. CBMs occur individually at the *N* or *C* terminus or multiply as tandem repeats at internal regions of a variety of enzymes including GA, amylase, pullulanase, water dikinase, and starch synthase (Figure S1) [7], [8], [9]. SBDs have been identified in members of ten different CBM families (CBM 20, 21, 25, 26, 34, 41, 45, 48, 53, and 58), and structural information is available in the RCSB Protein Data Bank (http://www.rcsb.org/) for only eight of these, families 20 [10], 21 [11], [12], 25 [13], 26 [13], 34 [14], 41 [15], 48 [16] and 58 [17]. These structures all show a characteristic β-sandwich fold. CBMs contain on average 100 amino acid residues and promote the interaction between the substrate and the enzyme, which in turn increases local substrate concentration at the active site of the catalytic domain [5]. This process aids the enzyme to degrade insoluble polysaccharides and target complex substrates like the type II blood group antigen H-trisaccharide [18], which resides within extracellular glycans [19]. Sugar-degrading enzymes lacking CBMs have significantly lower GH activities towards insoluble, but not soluble polysaccharide substrates [20].

Stacking of a CBM’s aromatic residues against sugar rings of polysaccharide or oligosaccharide ligands [21] and hydrogen bonding between polar residues and hydroxyl groups of carbohydrates [22] are crucial for CBM-glycan interactions. The crystal structure of *Ro*SBD shows that it is comprised of eight antiparallel β-strands forming two major β-sheets with a distorted barrel structure. It shows a conventional β-sandwich fold and immunoglobulin-like architecture, characteristic of most CBMs [23]. *Ro*SBD has two specific ligand-binding sites: site I (conserved in many starch-binding CBMs), consisting of W47, Y83, and Y94, and site II, consisting of Y32, F58 and Y67 [12], [24]. In addition, polar residues in site I (N50, N96, and N101) and site II (N29, K34, and E68) are involved in direct hydrogen bonding with ligands [12]. Interestingly, two polyN loops near site I (N46, N48, N49, N50 and N51 in loop β34, and N96, N97, N98 and N101 in loop β78) facilitate polysaccharide recognition *via* additional inter- and intramolecular hydrogen bonding. This feature is present in only three CBM21 members: *Ro*SBD, *Mucor circinelloides* SBD (*Mc*SBD), and another SBD from *R. oryzae* strain 3.2893 [12]. It appears that the overall structures of the unliganded (apo) and liganded *Ro*SBDs are identical except for the side-chain orientation of Y32 [12]. Upon binding maltoheptaose (abbreviated as G7), Y32 flips outward to form a binding “clamp” with F58 *via* aromatic ring stacking on both sides of the third glucose unit (Glc III) in the ligand.

General enzymatic hydrolysis of polysaccharides begins with CBM binding to a substrate to form a catalytic complex [25], which may alter substrate structures. For example, cellulose binding domain from *Cellulomonas fimi* endoglucanase A disrupts the structure of cotton fibers by sloughing off cellulose without covalent attachment and uncovering the ends of cellulose chains [26], and the SBD of *Aspergillus niger* GA (*An*SBD) disrupts the starch surface by twisting starch strands apart [5]. Such structural disruption allows greater substrate accessibility to enzymes [27] and increases the catalytic rate. The detailed mechanism of such action has not been elucidated, however.

To understand the function of CBMs, *Ro*SBD was used to investigate the modes of ligand binding and structure disruption. The natural ligands of *Ro*SBD are polysaccharides with α-1,4-glucopyranosidic linkages in a left-handed single helical conformation, or in parallel left-handed double helices containing approximately six glucose units per turn with a diameter of 1.3 nm [28]. This special structural feature allows various low molecular weight compounds, such as three I_2_ molecules and hemicyanine dyes, to get trapped in the amylose helical cavity, thereby forming inclusion complexes [29], [30]. Here, we monitored changes in the deep blue amylose-iodine complex as an indication of the disruption of the helical amylose structure in the presence of *Ro*SBD. Furthermore, molecular interactions between long-chain amylose and recombinant *Ro*SBD were monitored by atomic force microscopy (AFM), a method for measuring surface properties of biological macromolecules [31], [32]. In addition, isothermal titration calorimetry (ITC) was used to directly measure the binding affinity (*K*_a_) of the *Ro*SBD-glycan interaction in solution.

## Results and Discussion

### *Ro*SBD Alters the Ultrastructure of Amylose

**Figure 1:**
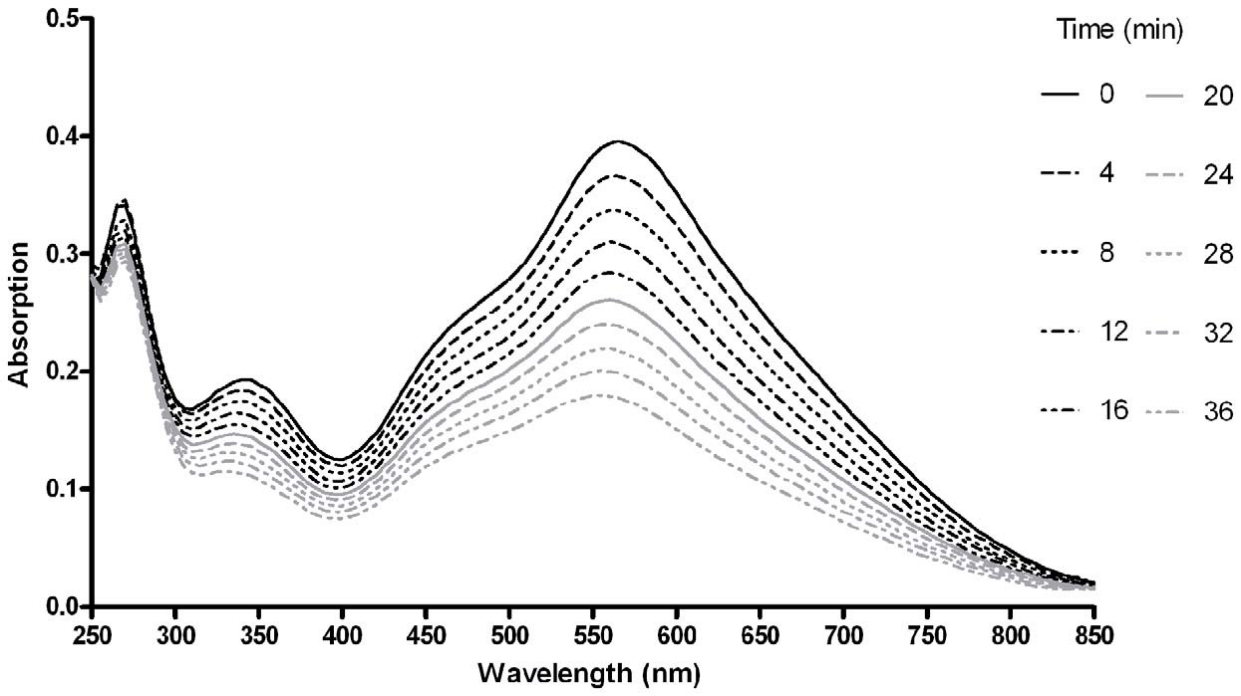
Disruption of the amylose-iodine complex by *Ro*SBD as a function of time. Absorption spectra were monitored at various time points after introduction of 5 µM *Ro*SBD to the amylose-iodine solution. The time points from up to down were 0, 4, 8, 12, 16, 20, 24, 28, 32, and 36 minutes.

An I_6_ unit composes of three I_2_ molecules, with an intramolecular distance of 3.0 Å between I_2_ molecules, can be accommodated inside the helix formed by amylose to give a deep blue color [29]. Owing to this shorter distance between I_2_ molecules compared to that of free I_2_, charge is delocalized along the iodine chain resulting in UV-visible absorption at approximately 600 nm [33]. An amylose-iodine solution containing amylose EX-I with an average degree of polymerization (DP) of 17 was mixed with I_2_ to react prior to the addition of *Ro*SBD. The effect of *Ro*SBD on the structural change of EX-I was measured by periodically monitoring absorption between 250 nm and 850 nm. Figure 1 showed a characteristic peak at 570 nm owing to iodine inside the amylose helical cavity. The absorption spectrum was similar to the published spectrum [34], but the λ_max_ slightly shifted to 570 nm, perhaps due to shorter amylose used here [35]. With increased incubation time from 0 to 36 min, the specific absorption at 570 nm decreased, indicating unstable amylose-iodine complex due to disruption of the amylose ultrastructure by *Ro*SBD.

**Figure 2:**
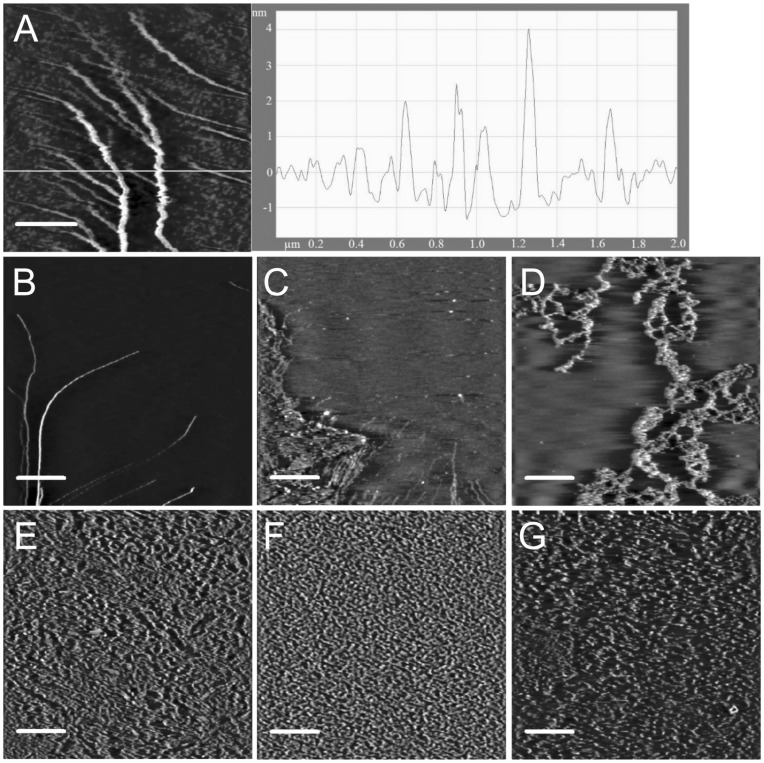
AFM images showing interactions between *Ro*SBD and amylose. (A) AFM images of amylose type III were obtained after depositing 5 µg/ml amylose at 25°C. The height of each fibril in the AFM image along the white line is shown in right panel of the graph. AFM images were obtained after incubating amylose type III solution with *Ro*SBD solution at 25°C for 16 h at various concentrations of amylose type III and protein, as follows: (B) 2.5 µg/ml, 30 nM; (C) 2.5 µg/ml, 300 nM; (D) 2.5 µg/ml, 3 µM; (E) 2.5 µg/ml, 30 µM; (F) 25 ng/ml, 30 µM; (G) 250 ng/ml, 30 µM. Scan sizes: (A) 2 µm × 2 µm; (B–G) 5 µm × 5 µm. Scale bars: (A) 500 nm; (B–G) 1 µm.

Since GA is able to degrade insoluble amylose, we investigated the effect of *Ro*SBD binding on the ultrastructure of insoluble amylose using AFM. We used amylopectin-free amylose type III that was extracted from potatoes and had an average DP of 900 [36], [37]. The AFM images of a 5 µg/ml amylose type III stock solution in water, recorded on a 1.5 cm × 1.5 cm piece of mica, was displayed in Figure 2A. The thickness of amylose type III shown in the right panel was estimated as 3.0 nm, approximately 6 times that of a single amylose chain (0.54 nm) [38], suggesting that the ultrastructure of amylose type III was assembled by several amylose fibrils. Interestingly, the ultrastructure of a 2.5 µg/ml amylose type III solution changed upon addition of increasing concentrations of *Ro*SBD (30 nM to 30 µM) from long linear fibrils (Figure 2B) to a net-like architecture (Figures 2C and 2D) and subsequently to short fibrils (Figure 2E). As the ratio of *Ro*SBD to amylose increased, the amylose fibrils became dendritic and exhibited extended chains. At high concentration of *Ro*SBD, assembled amylose fibrils were completely dispersed into short chains (Figures 2F and 2G). The appearance of amylose (at 2.5 µg/ml) significantly changed from a net-like architecture into short fibrils when the concentration of *Ro*SBD exceeded 3 µM. Thus, the *Ro*SBD concentration was fixed at 5 µM for all further experiments. It therefore appeared that *Ro*SBD bound to amylose and unwound the assembled amylose chains without enzymatic hydrolysis.

**Figure 3:**
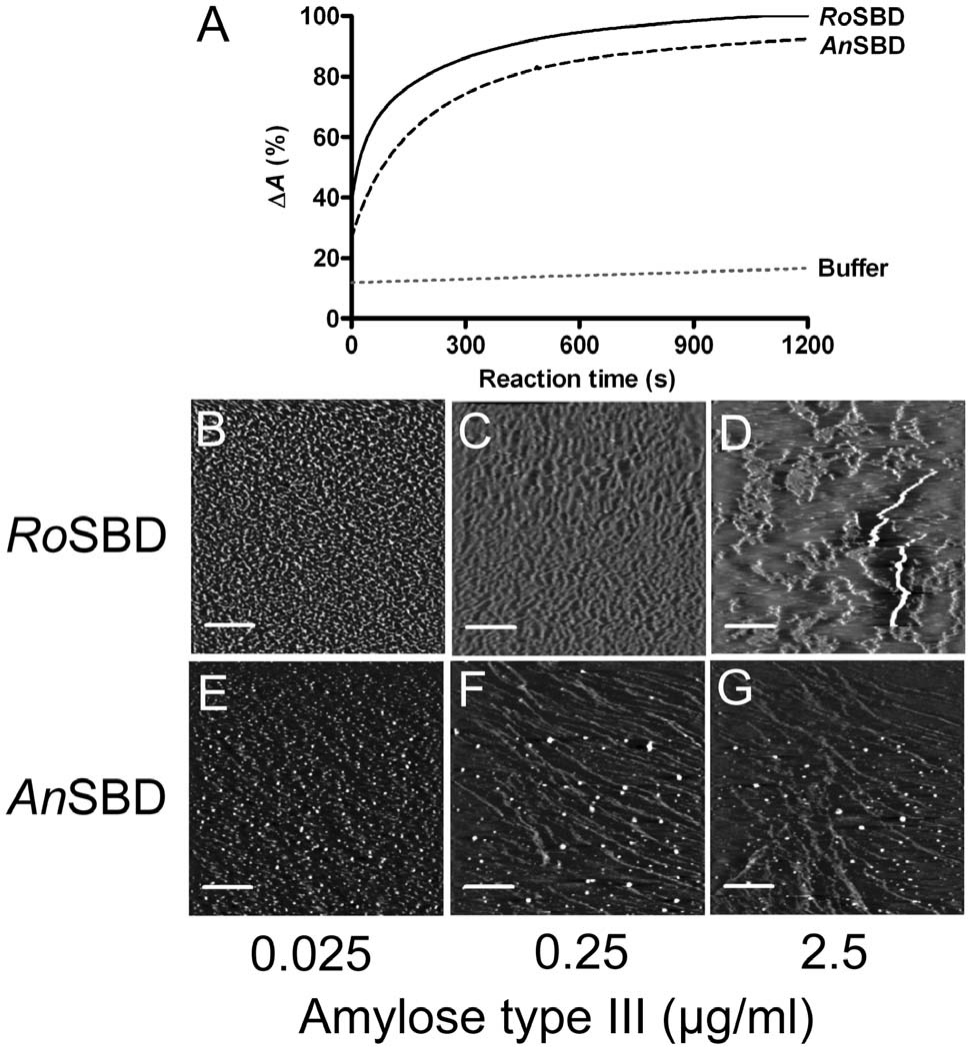
Change in morphology of amylose by SBDs. (A) The relative reduction in absorbance at 570 nm was recorded after adding 10 µM *Ro*SBD or *An*SBD to the amylose-iodine complex. (B–G) AFM images obtained after incubating amylose type III solution at the indicated concentration of amylose type III with 5 µM *Ro*SBD or *An*SBD at 25°C for 16 h. Scan size: 5 µm × 5 µm; scale bar: 1 µm.

GAs from *R. oryzae* and *A. niger* are widely used in the starch processing industries because of their remarkable thermal stability and their activity at nearly neutral pH [39]. Here, recombinant WT *Ro*SBD and WT *An*SBD were examined to understand the correlation between amylose disruption and the sugar binding activities of CBM21 and CBM20. To make a direct comparison between the amylose interaction with *Ro*SBD and *An*SBD, a 100-µl amylose-iodine mixture containing 0.2 mM amylose EX-I and 250 µM I_2_ in 50 mM sodium phosphate buffer (pH 7.4) was allowed to react at 25°C for 10 min prior to the addition of 10 µM *Ro*SBD or *An*SBD. The resulting decrease in absorbance at 570 nm was calculated and plotted against reaction time, and that of buffer alone was monitored as a negative control. Figure 3A showed that absorption of amylose-iodine decreased in the presence of *Ro*SBD or *An*SBD, suggesting that *An*SBD, like *Ro*SBD, also induced structural alteration of amylose.

The disruption of the amylose ultrastructure in the presence of *Ro*SBD or *An*SBD was further investigated by AFM. A progressive increase in amylose concentration (from 25 ng/ml to 250 ng/ml to 2.5 µg/ml) in the presence of 5 µM *Ro*SBD (Figures 3B to 3D) yielded complex loose spherical and net-like structures, presumably owing to incomplete unwinding of amylose by the limited amount of *Ro*SBD. Figures 3E to 3G showed quite different amylose architectures in the presence of 5 µM *An*SBD. At low amylose concentration, its architecture in the presence of *Ro*SBD was short fibrils (∼1.2 nm), but that of *An*SBD was short fibrils with tiny granules (∼0.75 nm). These differences implied that although *An*SBD disrupted the ultrastructure of amylose, its mechanism differed from that of *Ro*SBD. Elevated amylose concentration (250 ng/ml) resulted in lesser disruption of the long fibrils in the presence of *An*SBD, but not *Ro*SBD, indicating that *Ro*SBD disrupted amylose fibrils more effectively. The AFM images of amylose in the presence of *An*SBD differed from the circular structure of pea starch granules reported by Giardina *et al*. [25]. The mode of disruption and the consequent product varied with different SBDs and amylose sources as well as different methods of sample preparation. The ultrastructure of amylose was also clearly altered by *An*SBD, however, albeit *via* a different mechanism from that of *Ro*SBD.

### Two Unique Ligand-binding Clamps in *Ro*SBD Play a Key Role in Amylose Disruption

**Figure 4:**
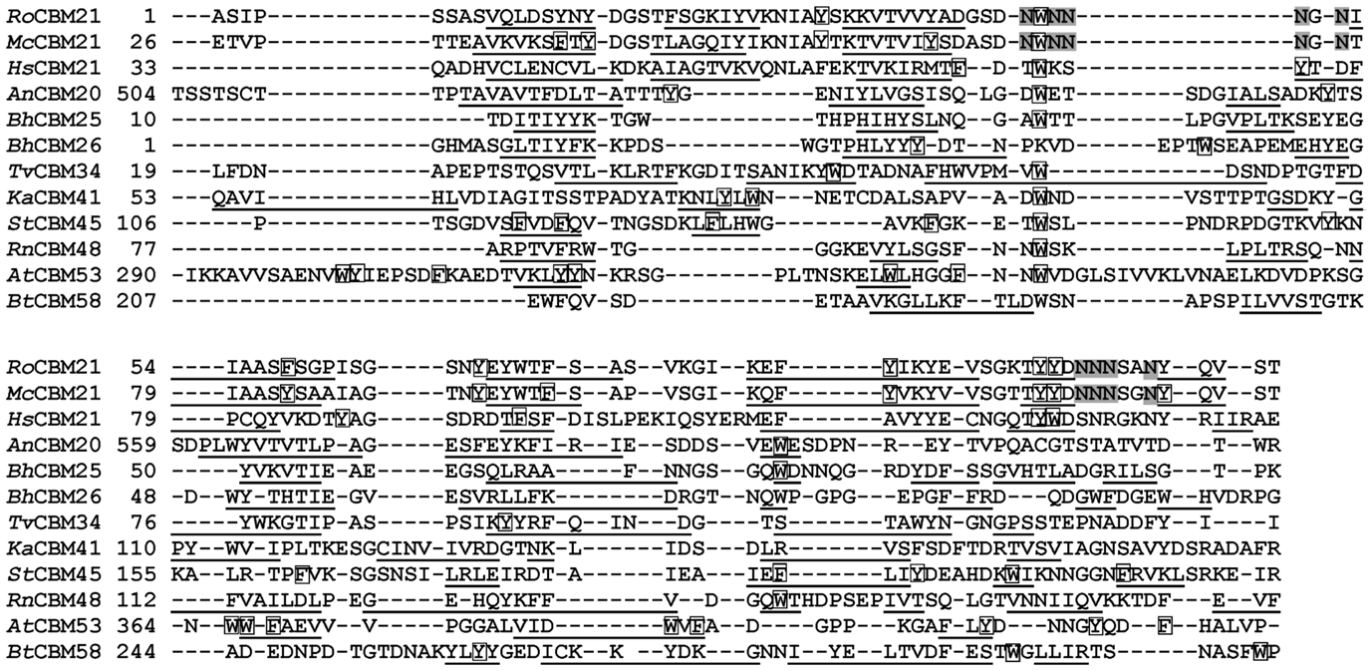
Sequence alignment of various starch-binding CBMs. Reported and FIA-predicted β-sheets of representative starch-binding CBMs are underlined; ligand-binding aromatic residues and two polyN loops, validated *in vitro*, are denoted by white and gray boxes, respectively.

Sequence alignment and homology modeling for CBMs is somewhat limited owing to low sequence identities (generally <25%); still, how amylose binding to *Ro*SBD differs from that of other SBDs could be garnered by multiple sequence analysis. Compared with other alignment methods [40], feature-incorporated alignment (FIA) algorithm developed in our laboratory [41] afforded better sequence matching amongst CBM family members by integrating conserved secondary structure elements and hydrophilic aromatic residues (HARs)–*i.e.*, characteristic aromatic residues flanked by polar residues residing within two adjacent neighboring amino acids [42]. Figure 4 showed the results of FIA analysis of representative SBDs from 10 starch binding CBM families. Because no 3D structural information is available for *Mc*SBD, CBM45 and CBM53, the FIA algorithm predicted secondary structural elements and HARs for these proteins and HARs for *Homo sapiens* CBM21 (*Hs*CBM21). Major secondary structural elements (underlined residues in Figure 4) aligned well in terms of relative positions and length, and putative functional HARs (highlighted residues) were found to be conserved. The FIA analysis also showed that three CBM21 members, *Ro*SBD, *Mc*SBD, and another SBD from a different *R. oryzae* strain 3.2893, each possessed two unique polyN loops near the HARs. In *Ro*SBD, these two loops, each located on one side of the sugar molecule, participate in ligand binding and act as a pair of clamp that interact with the hydroxyl groups of G7 [12]. Upon ligand binding, Y32 and F58 form the other pair of clamp. The presence of such dual binding clamps was unique to *Ro*SBD and rare in other CBM family members. We therefore investigated the function and importance of these two types of ligand-binding clamps of *Ro*SBD.

**Figure 5:**
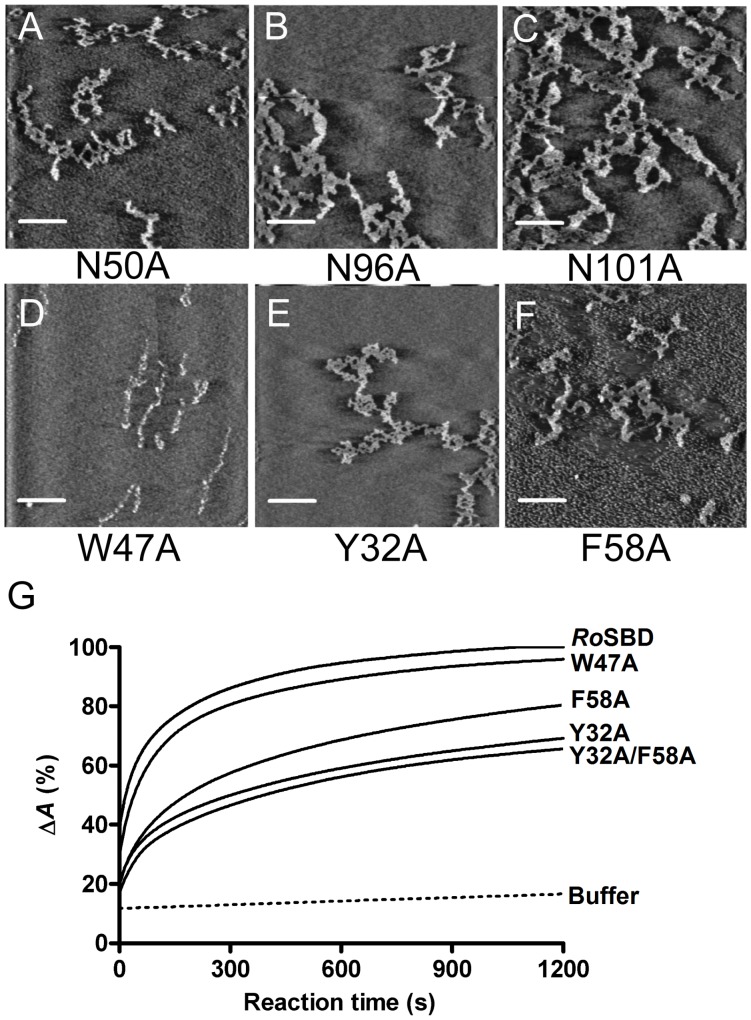
Change in morphology of amylose by *Ro*SBD mutants. (A–F) AFM images obtained after incubation of 25 ng/ml amylose type III solution with 5 µM of the various *Ro*SBD mutants at 25°C for 16 h. Scan size: 5 µm × 5 µm; scale bar: 1 µm. (G) The relative reduction in absorbance at 570 nm recorded after adding 10 µM mutants to the amylose-iodine complex.

Previous studies have indicated that several key HARs of *Ro*SBD, *i.e.*, Y32, W47, F58 and residues in the polyN loops, participate in ligand binding [12], [24], and we suggest that they may initiate amylose disruption. In this study, single-point mutations at N50, N96, and N101, each located on the surface of the polyN clamp, resulted in incomplete disruption of assembled amylose fibrils at low ligand concentration (25 ng/ml) (Figures 5A to 5C). It has been suggested that the polyN clamp serves to adjust ligand orientation [12]; here, we found that when key residues in the polyN clamp were mutated, ligand-binding affinity decreased and amylose structure disruption significantly lessened. These findings strongly suggested that the polyN clamp was important for amylose disruption.

The crystal structure of *Ro*SBD-G7 shows that W47 and Y32 of two different *Ro*SBDs cooperatively bind to the same G7 molecule. In addition, compared to unbound *Ro*SBD, Y32 flips outward to protrude from the surface and stacks on one side of G7, with F58 (from the same *Ro*SBD) stacking on the other side [12]. Mutation of each key functional HAR in turn led to decreased amylose binding and a significant reduction in amylose disruption–thus incomplete unwinding of amylose fibrils (25 ng/ml amylose type III) was observed for the mutant *Ro*SBDs W47A (Figure 5D), Y32A (Figure 5E) and F58A (Figure 5F). Upon addition of each mutant *Ro*SBD, the absorption spectrum of the amylose-iodine complex showed that the disruptive effects differed for each mutant. Figure 5G showed the reaction slopes of progressively decreasing magnitude for WT *Ro*SBD, W47A, F58A, Y32A and Y32A/F58A, indicating that the influence in disruption was the most significant when the Y32/F58 clamp was inactivated by double mutation; moreover, between these two residues, mutation of Y32 had a more pronounced effect. Interestingly, mutation at W47 only weakly diminished the complex disruption activity toward soluble ligand, strongly suggesting that the two ligand-binding sites in *Ro*SBD had different functions.

**Figure 6:**
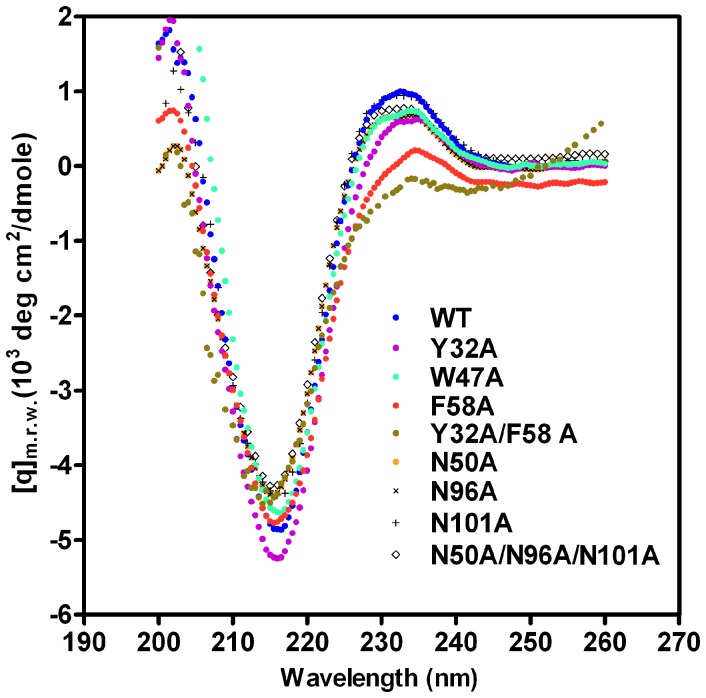
Far-UV CD spectra of WT and mutant *Ro*SBDs showing that the secondary structure has a β-sheet conformation. Spectra were obtained for WT and mutant *Ro*SBDs in 1 mM sodium acetate, pH 5.5, at 25°C. m.r.w., mean residue weight.

To confirm that the decreased amylose disruption and ligand-binding affinity of mutant *Ro*SBDs were indeed caused by the loss of a functional residue rather than conformational changes, the secondary structures of mutant *Ro*SBDs were analyzed by CD spectroscopy. The CD spectra of WT and mutant *Ro*SBDs (Figure 6) showed a minimum mean residue ellipticity at 215 nm, characteristic of the β-strand conformation. Thus, point mutations at these key ligand-binding residues did not appear to change the secondary structure of *Ro*SBD, as judged by CD spectroscopy. This strongly supported the conclusion that these key residues had a functional rather than structural role in amylose disruption.

Table 1 listed ITC-determined dissociation constants (*K*_d_) for the binding of soluble oligosaccharides to *Ro*SBDs. Reduced affinity for the mutants was shown as the percentage increase of *K*_d_ compared to that of WT *Ro*SBD. It has been reported that W47 plays a critical role in insoluble starch binding [12]. We observed that the mutant W47A *Ro*SBD showed a 68% reduction in binding affinity for corn starch compared to WT *Ro*SBD. The binding affinity of W47A for the other soluble polysaccharides decreased to a lesser extent however, with reductions of 45%, 53%, and 20% for βCD, G7, and amylose EX-I, respectively, compared with WT *Ro*SBD. Other residues near W47, such as those involved in the polyN clamp, also appeared to be involved in ligand binding–thus the polyN mutant (N50A/N96A/N101A) also showed lower *K*_d_ values, *i.e.*, decreases of 63%, 55% and 64% for βCD, G7, and amylose EX-I, respectively. Reduced binding affinity for βCD, G7, and amylose EX-I implied that ligand-binding affinity of site I in *Ro*SBD was a consequence of contributions by multiple residues, including a polyN clamp which played an important role in ligand binding, especially for long-chain polysaccharides.

**Table 1:** ITC-determined binding affinities between the recombinant WT and *Ro*SBD mutants and various polysaccharides.

	Protein	Dissociation constant, *K*_d_ (µM)		
	*Ro*SBD	βCD	G7	Amylose EX-I
	WT	20.7±2.3	197.2±27.7	42.7±2.9
Site I	W47A	37.9±2.7	413.2±10.3	53.4±3.6
	N50A/N96A/N101A	55.6±8.2	427.4±32.7	117.6±18.5
Site II	Y32A	334.4±100.3	1479.2±496.7	571.4±208.6
	F58A	98.0±31.7	657.9±214.3	229.4±31.5
	Y32A/F58A	354.6±79.5	1700.6±164.6	704.2±477.1

Regarding binding site II, the single-point mutants, Y32A and F58A, resulted in significantly weaker binding to all soluble polysaccharides tested compared to the W47A mutant. This indicated that site II participated mainly in soluble ligand binding. Of all of the single-point mutants, Y32A had the lowest binding affinity for the soluble polysaccharides, with a reduction in *K*_d_ of 94%, 87%, and 93% for βCD, G7, and amylose EX-I, respectively; this implied that Y32 was important for binding soluble ligands. Only moderately reduced binding affinity was observed for F58A, with a reduction of 79%, 70%, and 81% for βCD, G7, and amylose EX-I, respectively. The double mutant, Y32A/F58A, showed only a small drop in ligand-binding affinity compared with Y32A, strongly suggesting the binding affinity of this clamp was mainly contributed by Y32.

Our binding affinity data showed that the two binding sites of *Ro*SBD prefer different forms of polysaccharides and play different roles in ligand binding.

### Proposed Model for Molecular Interaction between Amylose and *Ro*SBD

We have characterized two binding clamps in *Ro*SBD: the polyN clamp near site I that facilitates sugar binding and stabilizes *Ro*SBD-ligand interactions to assist in amylose disruption, and the Y32/F58 clamp at site II, which stacks at the same glucose unit to form a stable binding complex. This special dual-clamp of *Ro*SBD significantly enhances the binding affinity for granular corn starch. In addition to binding starch, SBDs disrupt the structure of starch by twisting the amylose strands apart to expose more starch surface, allowing for further SBD binding [5]. This results in an increased apparent ligand-binding affinity and capacity. Differences between *Ro*SBD and other SBDs can be demonstrated by measuring *K*_d_ and *B*_max_ (the maximal amount of bound protein) values for *Ro*SBD binding to granular corn starch (*K*_d_ = 1.4±0.1 µM, *B*_max_ = 41.1±1.1 µmol/g) [12], which are higher than those for *An*SBD (*K*_d_ = 3.2±0.9 µM, *B*_max_ = 0.563±0.001 µmol/g) [43], *Bacillus halodurans* CBM25 (*Bh*CBM25; *K*_d_ = 30.3±2.8 µM, *B*_max_ = 0.53±0.03 µmol/g), and *Bh*CBM26 (*K*_d_ = 27.0±1.5 µM, *B*_max_ = 0.99±0.03 µmol/g) [13]. Our AFM data also revealed that when *Ro*SBD bound to amylose, conformational changes occurred to allow more amylose surface to be accessed for additional *Ro*SBD binding, thereby increasing binding capacity.

**Figure 7:**
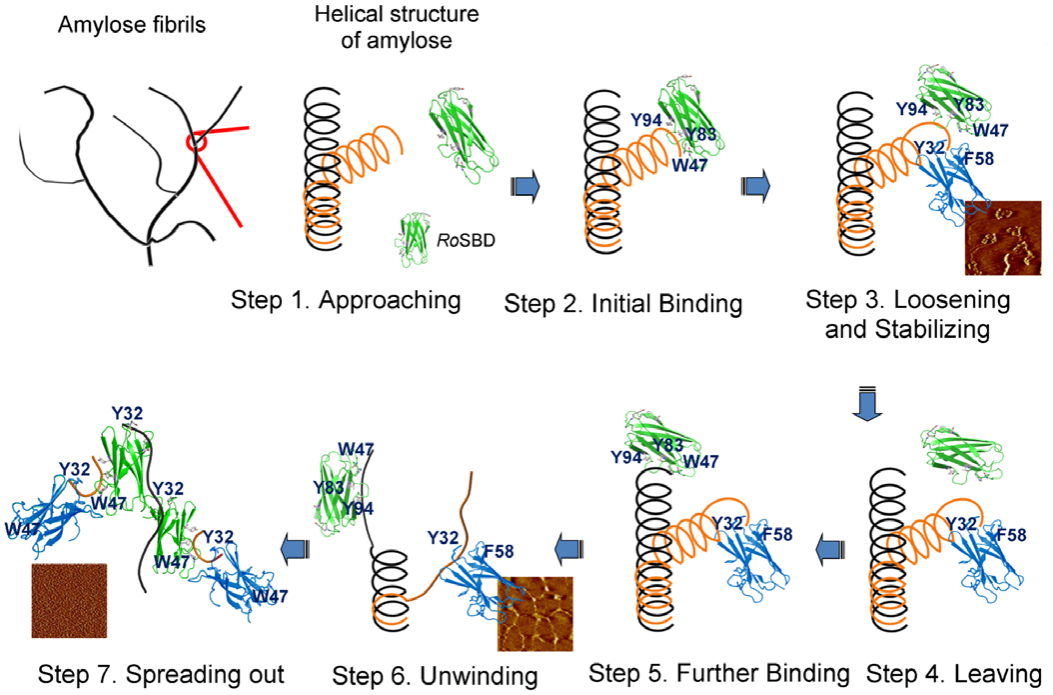
Proposed model for *Ro*SBD-mediated disruption of amylose. The molecular orientation of *Ro*SBD and amylose was built using the 3D crystal packing structure (PDB ID: 2V8M). Step 1: Approaching. *Ro*SBD approaches the amylose fibrils. Step 2: Initial Binding. *Ro*SBD binds to the non-reducing end of amylose *via* binding site I. Step 3: Loosening and stabilizing. *Ro*SBD-induced relaxation of amylose fibrils exposes more surface for binding, and a second *Ro*SBD molecule stabilizes the extended amylose structure that was unraveled by binding of the first *Ro*SBD molecule to non-reducing ends. Step 4: Leaving. The initial *Ro*SBD leaves the extended amylose. Step 5: Further binding. The departing *Ro*SBD binds to the non-reducing end of another neighboring amylose and disrupts the ultrastructure. Step 6: Unwinding. The repeated disruption process results in the loosening and unwinding of assembled amylose fibrils to form smaller fibers. Step 7: Spreading out. *Ro*SBDs finally convert fibers into single amylose molecules and spreads them out.

Among three different starches, *Ro*SBD showed the strongest binding affinity for βCD (*K*_d_ = 20.7±2.3 µM) and higher binding affinity for amylose EX-I (DP17, *K*_d_ = 42.7±2.9 µM) than G7 (*K*_d_ = 197.2±27.7 µM). The structural unit of βCD is seven glucose units in a helical conformation, with a diameter of 1.53 nm [44] resembling the helical structure of amylose. G7 is composed of seven linear glucose units, resembling uncoiled amylose disrupted by *Ro*SBD at the terminal end. Amylose EX-I is composed of 17 glucose units, containing approximately 3 helical turns, more closely resembling native amylose at the terminal end of its long chain but with a relaxed structure. Based on our results, we propose that *Ro*SBD first binds to helical amylose, owing to its strongest binding affinity for βCD. Following the resulting conformational changes, binding between *Ro*SBD and the now more relaxed amylose becomes weaker to allow *Ro*SBD to dissociate from the amylose. Based on the binding affinity and disruption phenomenon of amylose, we propose a model of molecular interaction for *Ro*SBD (Figure 7). The molecular orientation of *Ro*SBD and amylose was built using 3D crystal packing data (PDB ID: 2V8M) [12]. Because the two binding sites of *Ro*SBD are topologically distant, it is hard to accomplish the process of ligand binding, disruption, and stabilization with only one molecule of *Ro*SBD; indeed the crystal structure of *Ro*SBD-G7 shows that two molecules of *Ro*SBD bind to the same G7. We therefore envision that two molecules of *Ro*SBD coordinate in the overall process of unwinding amylose. Initially, one *Ro*SBD molecule approaches the amylose fibril and binds strongly to the helical wheel of the amylose chains. Upon amylose binding, the hydrophobic curvature of the binding site I (W47, Y83, and Y94) and the hydrophilic polyN clamp alters the amylose ultrastructure. The helical structure is thereby relaxed, allowing binding of the second *Ro*SBD molecule to the non-helical end of amylose *via* its Y32/F58 clamp at site II. Next the initially bound *Ro*SBD dissociates owing to reduced binding affinity for the relaxed amylose conformation, and this *Ro*SBD then shifts to bind to adjacent non-reducing ends [3] to further uncoil the amylose. This process exposes more polysaccharide surface for binding of additional *Ro*SBDs. The repeated disruption results in the loosening and unwinding of the assembled amylose fibrils to form shorter fibers.

### The Y32/F58 Ligand-binding Clamp is Conserved in CBM20 and CBM21

*An*SBD has a similar structure to *Ro*SBD, with two binding sites that are thought to play different roles [25], [45]: one binding site, W590 (which corresponds to site I of *Ro*SBD), is responsible for the initial recognition of starches; the other binding site, W560, binds the ligand tightly [25]. Some SBDs, however, such as CBM25 and CBM26 [13], CBM 45 [46], and CBM 53 [47], have only a single ligand-binding site, but they comprise a tandem repeat of individual CBMs. Such tandem repeats could augment the low ligand-binding affinity of only one ligand-binding site. For instance, the binding affinity of CBM25 fused to CBM26 (CBM25/26) for granular corn starch (*K*_d_ = 0.63±0.08 µM) is much higher than that of either CBM25 (*K*_d_ = 30.3±2.8 µM) or CBM26 (*K*_d_ = 27.0±1.5 µM) alone [13].

**Figure 8:**
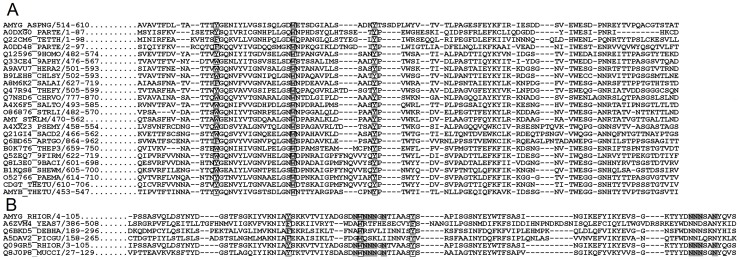
Sequence alignment of representative CBM20 and CBM21 members with three key aromatic ligand-binding residues. No pair of sequences shares higher than 80% identity. The reported ligand-binding residues and polyN loops of (A) CBM20 (alignment template: *An*CBM20, AMYG_ASPNG) and (B) CBM21 (alignment template: *Ro*CBM21, AMYG_RHIOR) and their sequence similarity to other members are highlighted in white and gray boxes, respectively.

**Figure 9:**
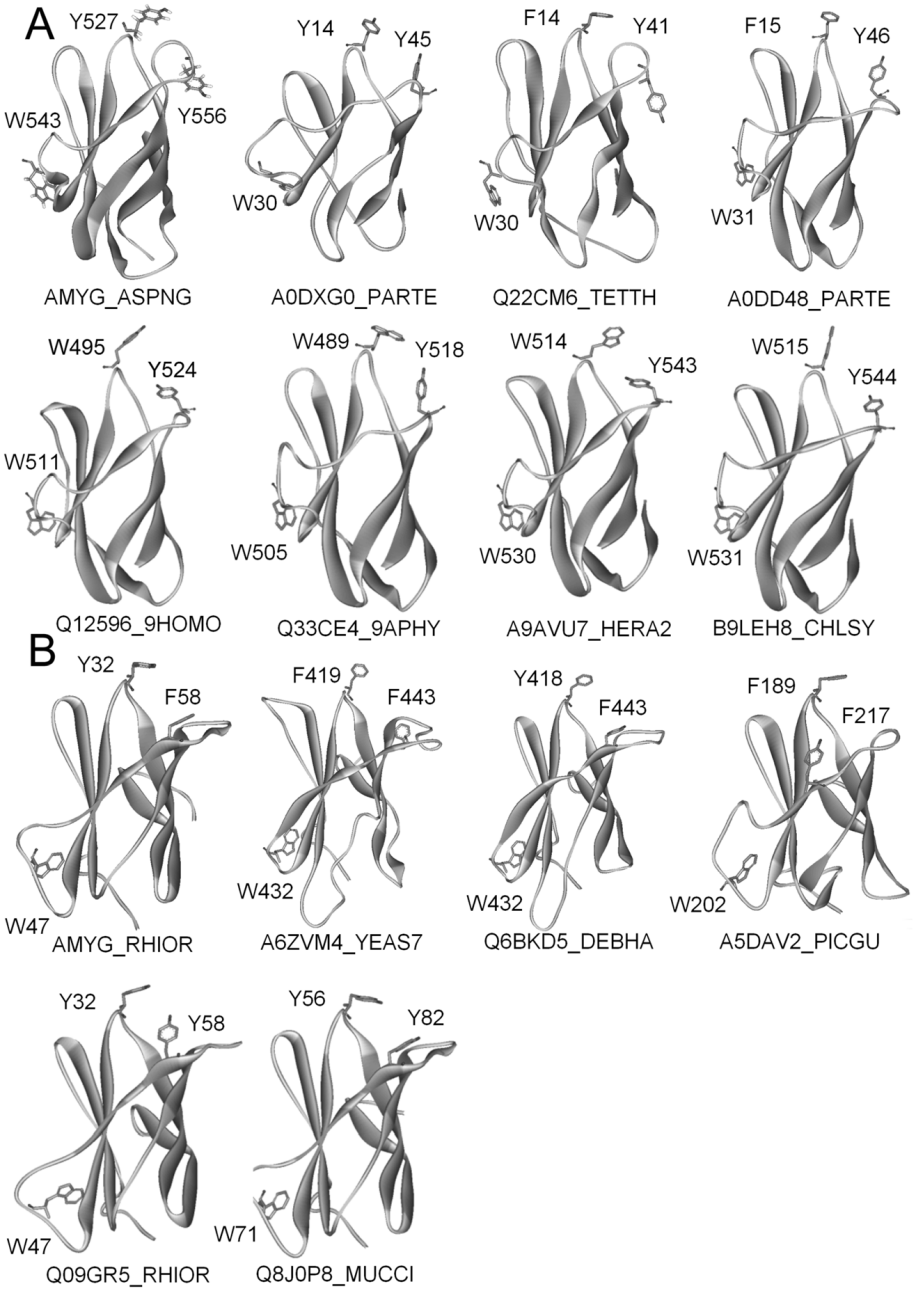
Ribbon diagrams showing the tertiary structures of representative CBM20 and CBM21 family members with three key ligand-binding aromatic residues. (A) CBM20. (B) CBM21. Conserved residues are shown in stick representation. The structures of AMYG_ASPNG and AMYG_RHIOR were experimentally determined, and all others were simulated using FIA-based homology modeling.

It is known that Y32, W47, and F58 in *Ro*SBD are key residues for ligand binding [12]. We found that these residues are conserved at the corresponding positions of Y527, W543, and Y556 in *An*SBD, respectively (shown in grey in Figure 8A). Interestingly, these three key ligand-binding HARs were found to be highly conserved in 23 members of the CBM20 family (Figure 8A) and 5 members of the CBM21 family (Figure 8B). Using FIA-based homology modeling [41], *in silico* structures of these CBM20 and CBM21 members were simulated using *An*CBM20 (AMYG_ASPNG, PDB ID: 1AC0) and *Ro*CBM21 (AMYG_RHIOR, PDB ID: 2V8L) as the template structures (shown in Figure 9 and Figure S2, respectively). As expected, Y527 and Y556 in *An*SBD form a similar binding clamp as Y32 and F58 in *Ro*SBD (Figure 9A AMYG_ASPNG). This binding clamp appears to be present in the loops of all CBM20 and CBM21 structures examined and strongly supports the notion that these CBM members bear differential binding properties in site I and site II, controlled by three conserved key ligand-binding HARs.

Multiple sequence alignment of 107 CBM20 and 76 CBM21 family members revealed that only *Ro*SBD, *Mc*SBD, and another SBD from a different *R. oryzae* strain (strain 3.2893) have this distinctive dual-clamp sequence, and few members of the CBM20 and CBM 21 families (23 of 107, and 3 of 76, respectively) possess a single binding clamp corresponding to binding site II of *Ro*SBD. Although the crystal structures of these CBM members with single binding clamps have not been solved, they are expected to show similar binding properties to *Ro*SBD. This result also conformed with previous study that CBM20 and 21 share a common evolutionary origin and belong to a CBM clan with similar tertiary structures, conserved catalytic machinery and reaction mechanism [48]. Interestingly, although CBM20 and 48 very possibly share a common ancestor, the Y32/F58 ligand-binding clamp is not present in CBM48 probably due to early divergence in evolution [8].

### Prospects of CBM Function and Engineering

The existence of CBMs in virulence factors and proteins involved in metabolism [49], [50] and in promoting tissue destruction and enhancing either bacterial spread or pathogenesis [18], [51] has also been demonstrated in recent reports. For examples, *Streptococcus pneumonia* and *Streptococcus pyogenes* are reported to recognize host tissue by pullulanases that contain a tandem CBM41 repeat. These CBMs assist *S. pneumonia* in binding to intracellular glycogen in alveolar cells for polysaccharide degradation by the pathogen [52]. Understanding the ligand-binding features and structure-disrupting mechanisms of CBMs may facilitate the design of new compounds to target CBM-associated pathogens and prevent their spread. Here, we report the first molecular mechanism of *Ro*SBD action on amylose structure disruption, with a detailed mechanism of interaction, and different functions of the two ligand-binding sites. Because CBMs are present in many different enzymes involved in a variety of processes, such as carbohydrate metabolism [53], [54], structure formation and degradation of plant materials [55], [56], immunological recognition [57], targeting [58], and antibiosis, this functional information provides a useful basis that may lead to new designs and applications for CBMs in industry.

## Materials and Methods

### Strains, Media, Plasmids and Chemicals

TOP10F′ *Escherichia coli* (Invitrogen) was used for plasmid manipulation, and *E. coli* strain BL21-Gold (DE3) (Novagen) was used for protein expression. *E. coli* cells harboring the plasmid vectors pGEM-T Easy cloning vector (Promega), pET23a (+), or pET15b (Novagen) were grown in Luria–Bertani medium (1% tryptone, 0.5% yeast extract, and 0.5% NaCl) containing 100 µg/ml ampicillin at 37°C. Chemicals including iodine, granular cornstarch, and amylose powder from potato starch (type III) were purchased from Sigma. Amylose EX-I with a DP of 17 was purchased from Hayashibara.

### Protein Expression and Purification

The DNA fragments encoding the SBD of *An*GA were amplified by PCR using primer sets, 5′-**CATATG**AGCAAGACCAGCACC-3′ (*An*SBD forward) and 5′-**CTCGAG**CCGCCAGGTGTCAGT-3′ (*An*SBD reverse), where the restriction sites are bolded. The PCR product was cloned into the pGEM-T Easy cloning vector (Promega) and verified by DNA sequencing (Mission Biotech Co., Ltd). The DNA fragment of *An*SBD was subsequently ligated into the pET15b expression vector at the *Nde*I and *Xho*I sites to generate pET15b-*An*SBD. Expression and purification of *An*SBD was carried out as for *Ro*SBD [59].

### Site-directed Mutagenesis

All single-point mutants of *Ro*SBD were generated and constructed as described [12] using PCR-based QuikChange site-directed mutagenesis (Stratagene). pET23a(+)-*Rosbdy32a* and -*Rosbdn50a* were used as templates for the double mutant Y32A/F58A and the polyN mutant N50A/N96A/N101A, respectively, and were amplified using *Pfu* Turbo DNA polymerase (Stratagene). The two complementary primers containing the desired mutations were: *Rosbdf58a* forward, 5′- CATCATTGCTGCTTCTGCCTCTGGCCCTATC-3′; *Rosbdf58a* reverse, 5′-GATAGGGCCAGAGGCAGAAGCAGCAATGATG-3′; *Rosbdn96/n101a* forward; 5′-CATACTATGATGCCAACAATTCTGCCGCTTACCAAGTATC-3′; *Rosbdn96/n101a* reverse, 5′-GATACTTGGTAAGCGGCAGAATTGTTGGCATCATAGTATG-3′. The sequences of the mutant plasmids were verified by Mission Biotech Co., Ltd., and constructs were then transformed into competent *E. coli* BL21-Gold (DE3) for protein expression.

### UV Spectrophotometry

Amylose EX-I powder (Hayashibara Biochemical Laboratories Inc.) was dissolved in 50 mM sodium phosphate buffer, pH 7.4, to give a concentration of 3 mg/ml (approximately 1 mM) and heated to 90°C with stirring for 1 h. The amylose EX-I solution was then diluted with sodium phosphate buffer to give a 0.2 mM working solution and cooled to 25°C. Iodine was dissolved in DMSO to give a concentration of 25 mM. Then a 25 mM I_2_ solution (1 µl) was added to 100 µl of the amylose EX-I working solution and incubated at 25°C for 10 min. After adding 5 µM *Ro*SBD, the absorption spectrum of the solution was monitored between 250 nm to 850 nm.

The change of absorption of the blue color of the amylose-iodine solution at 570 nm was monitored as a function of time after addition of 10 µM *Ro*SBD (or *An*SBD) to the amylose-iodine complex solution. As a control, the time-dependent decrease in 570 nm absorption of the amylose-iodine solution in the absence of any added protein was also monitored after addition of buffer alone. The time-dependent overall fractional change in absorption (Δ*A*) caused by incubating protein with the amylose-iodine solution was depicted as the percentage change of absorption at 570 nm.

### AFM

Amylose type III powder (Sigma-Aldrich Inc.) was dissolved in water at a concentration of 1 mg/ml and heated to 90°C with stirring for 1 h. The amylose type III solution was diluted to a 5 µg/ml working solution with water at 90°C. The solution was then slowly cooled to approximately 40°C, filtered using a 0.45-µm cellulose filter, and then further cooled to 25°C. To investigate the interactions between the recombinant proteins and amylose type III, various concentrations of each protein in 50 mM sodium acetate buffer, pH 5.5, were mixed with the amylose working solution and incubated at 25°C for 16 h. Samples for AFM were obtained by dropping 6 µl of the amylose solution, or the amylose-SBD solution, onto freshly cleaved mica and then air-dried at 25°C in a dust-free environment before scanning.

The AFM instrument was a 5500 AFM/SPM microscope (Agilent) equipped with V-shaped silicon nitride (Si_3_N_4_) cantilever probes with 0.08 Nm^–1^ spring constants. All images were captured at 512 × 512 pixel resolution and were processed using Scanning Probe Image Processor software v4.0 (Image Metrology).

### FIA and Structure Prediction

The following ten starch-binding CBMs were used for FIA and structure prediction because of their representative architectures and sequences; they are listed by *species of origin* (abbreviated name indicating family number, accession number from GenBank): *Aspergillus niger* (*An*CBM20, CAK38411), *Rhizopus oryzae* (*Ro*CBM21, ABB77799), *Mucor circinelloides* (*Mc*CBM21, AAN85206), *Homo sapiens* (*Hs*CBM21, BAB14811), *Bacillus halodurans* (*Bh*CBM25 and *Bh*CBM26, BAB04132), *Thermoactinomyces vulgaris* (*Tv*CBM34, BAA02471), *Klebsiella aerogenes* (*Ka*CBM41, AAA25124), *Solanum tuberosum* (*St*CBM45, CAA70725), *Rattus norvegicus* (*Rn*CBM48, AAC52579), *Arabidopsis thaliana* (*At*CBM53, AAD30251), and *Bacteroides thetaiotaomicron* (*Bt*CBM58, AAO78803). The X-ray crystallographic structures of *An*CBM20 (PDB ID: 1AC0) [10], *Ro*CBM21 (PDB ID: 2V8L) [12], *Hs*CBM21 (PDB ID: 2EEF), *Bh*CBM25 (PDB ID: 2C3V) [13], *Bh*CBM26 (PDB ID: 2C3G) [13], *Tv*CBM34 (PDB ID: 1JI1) [14], *Ka*CBM41 (PDB ID: 2FGZ) [15], *Rn*CBM48 (PDB ID: 1Z0M) [16] and *Bt*CBM58 (PDB ID: 3K8K) [17], as well as the predicted structures of *Mc*CBM21, *St*CBM45 and *At*CBM53, were used for multiple sequence alignment using FIA [41].

In total, 102 representative domains for the CBM20 family, and 76 for the CBM21 family, were collected from the Pfam database (http://pfam.sanger.ac.uk), with any pair having sequence identity <80% retained for further analysis. Using FIA-based homology modeling, *in silico* structures of these CBM20 and CBM21 members were simulated using *An*CBM20 (*An*CBM20/AMYG_ASPNG, PDB ID: 1AC0) and *Ro*CBM21 (*Ro*CBM21/AMYG_RHIOR, PDB ID: 2V8L) as template structures from the Database of Simulated Carbohydrate-Binding Module Structures (DS-CBM; http://dscbm.life.nthu.edu.tw/) [42].

### CD Spectroscopy

CD spectra were recorded on an Aviv CD spectrometer (model 202) equipped with a 450-W xenon arc lamp. Far-UV spectral analysis at 200–260 nm was performed in a rectangular quartz cuvette with a 0.1-cm path length at 25°C using a scan rate of 4 nm·s^−1^ and a bandwidth of 0.5 nm. Each spectrum was the average of three consecutive scans and was baseline-corrected by subtracting the spectrum of buffer alone at the same temperature.

### ITC

Carbohydrate–protein binding affinities were determined by ITC using a VP-ITC instrument (MicroCal Inc.). All samples were degassed extensively prior to the measurement. Reactions were carried out by titrating 25×3 µl aliquots of each polysaccharide solutions into 50 mM sodium acetate buffer in the ITC cell at 25°C, pH 5.5. For individual titrations, injections into the 1.4-ml sample cell containing 40 µM *Ro*SBD were made every 240 s using a computer-controlled microsyringe. Binding isotherms, corrected for the heat of dilution, were analyzed by non-linear regression using Origin v7.0 software (MicroCal) supplied with the ITC instrument. The fitted data yielded the *K*_a_; the *K*_d_ was obtained as the reciprocal of the association constant (1/*K*_a_).

## Supporting Information

Figure S1 **Typical architectures of starch-binding CBMs.** Reported SBDs located in the *N* or *C* terminus or in internal regions of representative enzymes containing CBMs 20, 21, 25, 26, 34, 41, 48, 53 and 58 (GenBank accession numbers and protein lengths are listed in parentheses). Black, gray and white boxes represent SBDs, other internal domains and catalytic domains, respectively. The position and size of CBMs and other functional domains correlate with the size of the full-length enzymes. Abbreviations used: GH, glycoside hydrolase family; GT, glycosyltransferase family.
(TIF)

Figure S2 **Ribbon diagrams of the tertiary structures of representative CBM20 family members with three key ligand-binding aromatic residues.** Conserved residues are shown in stick representation. The structures were simulated using FIA-based homology modeling.
(TIF)
