# Supplementary figures and images for: Two Unique Ligand-Binding Clamps of Rhizopus oryzae Starch Binding Domain for Helical Structure Disruption of Amylose
Source: PLoS One. 2012 Jul 17;7(7):e41131. doi: 10.1371/journal.pone.0041131 (PMC3398936; doi:10.1371/journal.pone.0041131)

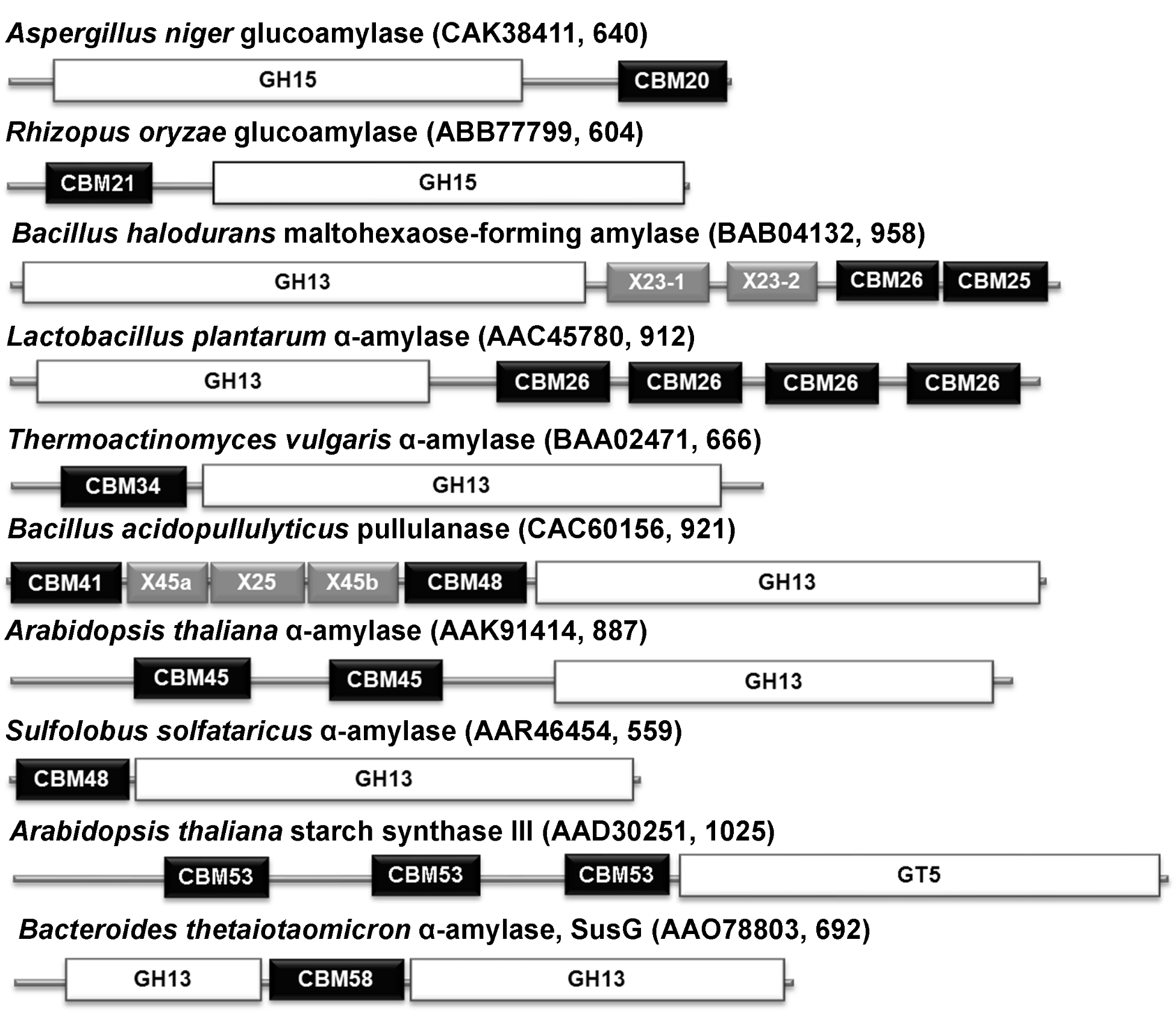

Supplement: Figure S1 — Typical architectures of starch-binding CBMs. Reported SBDs located in the N or C terminus or in internal regions of representative enzymes containing CBMs 20, 21, 25, 26, 34, 41, 48, 53 and 58 (GenBank accession numbers and protein lengths are listed in parentheses). Black, gray and white boxes represent SBDs, other internal domains and catalytic domains, respectively. The position and size of CBMs and other functional domains correlate with the size of the full-length enzymes. Abbreviations used: GH, glycoside hydrolase family; GT, glycosyltransferase family. (TIF) [file pone.0041131.s001.tif]

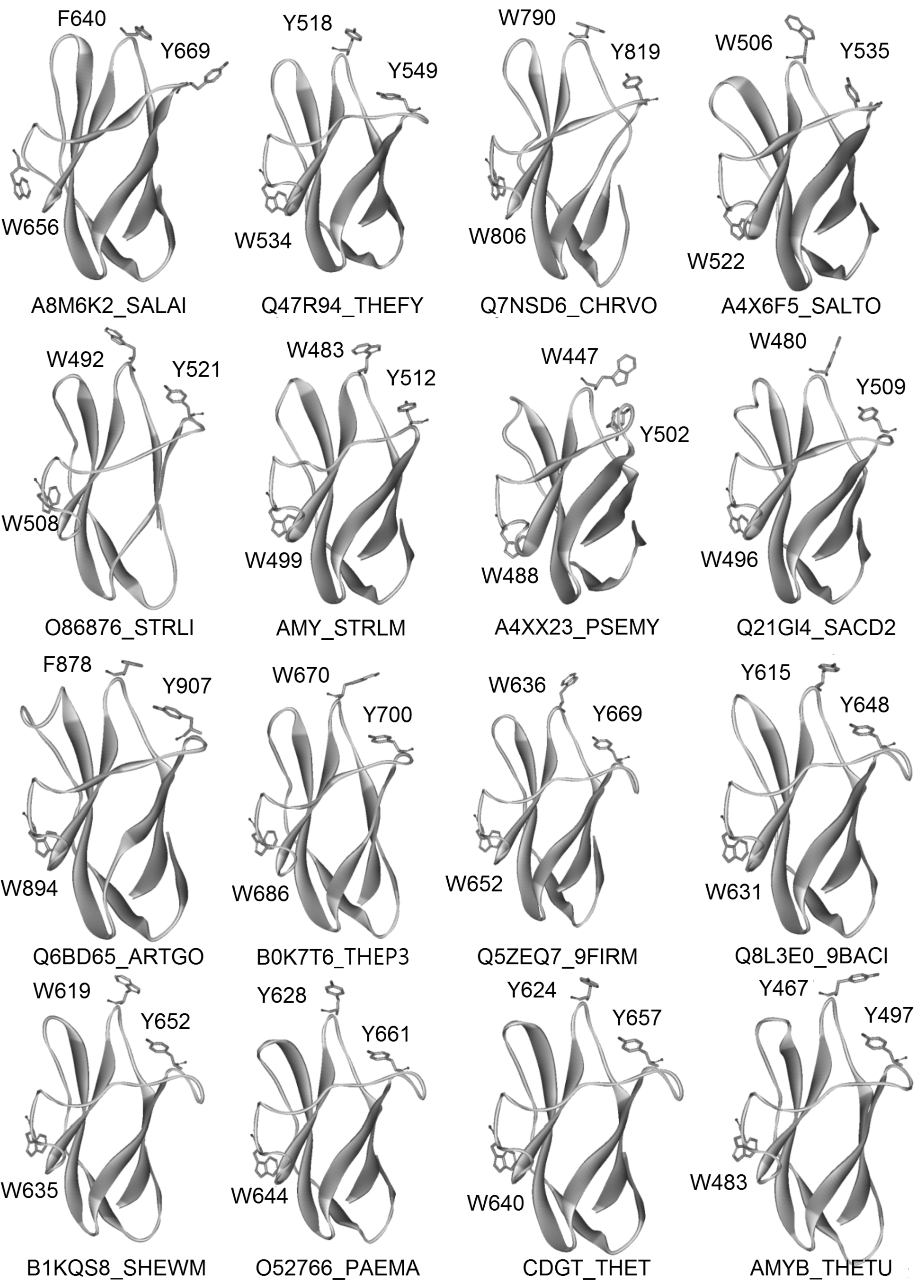

Supplement: Figure S2 — Ribbon diagrams of the tertiary structures of representative CBM20 family members with three key ligand-binding aromatic residues. Conserved residues are shown in stick representation. The structures were simulated using FIA-based homology modeling. (TIF) [file pone.0041131.s002.tif]
